# From fatal disease to functional cure: 25 years of tyrosine kinase inhibition in chronic myeloid leukemia

**DOI:** 10.1038/s41375-026-02984-5

**Published:** 2026-05-22

**Authors:** Andreas Hochhaus, Moshe Talpaz, Giuseppe Saglio, Jorge E. Cortes, Timothy Hughes, Jane Apperley, Oliver Hantschel, Delphine Rea, Peter Schuld, Hagop Kantarjian

**Affiliations:** 1 Klinik für Innere Medizin II, Hematology/Oncology, Universitätsklinikum Jena and Comprehensive Cancer Center Central Germany, Campus Jena, Jena Germany; 2 Division of Hematology-Oncology, University of Michigan Rogel Cancer Center; Internal Medicine, University of Michigan, Ann Arbor, MI USA; 3 Department of Clinical and Biological Sciences, University of Turin, Torino, Italy; 4 O’Neal Cancer Center, University of Alabama at Birmingham, Birmingham, AL USA; 5 Precision Cancer Medicine Theme, South Australian Health & Medical Research Institute, Adelaide, and Adelaide University, Adelaide, SA Australia; 6 Centre for Haematology, Imperial College London, Hammersmith Hospital, London, and Department of Haematology, Hammersmith Hospital, London, UK; 7 Institute of Physiological Chemistry, Faculty of Medicine, Philipps University of Marburg, Marburg, Germany; 8 Département d’Hématologie, Hôpital Saint-Louis, APHP, Paris, France; 9 Novartis Pharma AG, Basel, Switzerland; 10 Leukemia Department, University of Texas MD Anderson Cancer Center, Houston, TX USA

**Keywords:** Drug development, Therapeutics

## Abstract

Chronic myeloid leukemia (CML) is a myeloproliferative neoplasm characterized by the presence of the Philadelphia chromosome, which results from a translocation between chromosomes 9 and 22. This alteration gives rise to the *BCR::ABL1* fusion oncogene, whose constitutive tyrosine kinase activity promotes uncontrolled proliferation and survival of leukemic cells. The discovery of this molecular driver laid the foundation for the development of tyrosine kinase inhibitors (TKIs), which transformed CML management from treatment with non-specific modalities to establishing a new paradigm for precision oncology with targeted therapies. Imatinib, the first TKI, demonstrated unprecedented hematologic, cytogenetic, and molecular responses, rapidly becoming the standard of care in CML. Subsequent generations of TKIs were developed to overcome resistance and intolerance to imatinib. These advances improved survival in CML, bringing life expectancy close to that of the general population for the patients who respond to treatment, and shifting treatment goals towards quality of life and the possibility of treatment-free remission (TFR). Landmark discontinuation studies showed that approximately half of eligible patients can maintain TFR. Despite these achievements, important challenges remain, including TKI resistance, safety considerations, and the relatively low proportion of patients who achieve and maintain TFR. Newer-generation TKIs, combination strategies, immunomodulatory approaches, and emerging treatment modalities such as degraders offer promising avenues to further improve outcomes, expand TFR eligibility, ensure optimal quality of life, address resistance mechanisms, and render therapy available and affordable to all patients with CML worldwide.

## Introduction

Few drugs in the history of oncology have shown a level of success similar to that of the BCR::ABL1 tyrosine kinase inhibitors (TKIs) in chronic myeloid leukemia (CML). From what was a lethal disease before 2000, CML was transformed into a chronic illness, with a 10-year relative survival rate of 92% [1]. TKIs caused the treatment paradigm of CML to shift from non-specific approaches to directly and selectively targeting the molecular driver of disease [2, 3], a concept with lasting legacy for the treatment of cancer and other diseases. The ability of TKIs to improve CML outcomes resulted in a continuous evolution of diagnostics and monitoring, with techniques that allowed for ever lower limits of residual disease detection rising in parallel with advances in treatment. With responses getting deeper, the goals of treatment moved from extending overall survival (OS) to achieving early responses [4], improving quality of life (QoL), and achieving treatment-free remission (TFR) [5]. The advent of TKIs also led to the standardization of diagnostics and response assessment in cancer [6], ushering in the era of precision oncology [2]. The biological knowledge around the disease made it possible to understand the mechanisms of resistance as they arose and how to overcome them. Molecular alterations in different cancer types became the focus of research and led to the development of targeted drugs [7], thereby improving specificity and reducing the burden of treatment-associated side effects.

CML is a hematological malignancy driven by abnormal growth of myeloid cells. Chronic phase is the earliest and most common disease stage; if untreated, CML progresses to advanced phase, usually a blast phase which resembles acute myeloid leukemia (AML) or acute lymphoblastic leukemia (ALL) [8]. Some clinicians and pathologists also consider an intermediate phase termed accelerated phase. Before TKIs, treatments for CML were largely ineffective, with the exception of allogeneic hematopoietic stem cell transplantation (HSCT). Conventional cytotoxic agents such as busulfan and hydroxyurea controlled peripheral blood counts but did not prevent progression to advanced disease [9, 10], leading to 10-year survival rates of approximately 10–20% [11, 12]. From the 1980s, interferon alpha became the mainstay of CML treatment, marginally improving survival and cytogenetic response [10, 13, 14]. Indeed, interferon alpha was the first therapy that demonstrated the value of eliminating (or at least reducing) malignant leukemic clones through induction of cytogenetic responses. However, the main limitation of interferon alpha treatment was its overall poor response rate, with molecular studies using the novel reverse transcriptase–polymerase chain reaction (RT-PCR) revealing the persistence of disease in most patients post-treatment [15]. In the 1980s, allogeneic HSCT was established as the only curative option for CML [16]. However, due to limitations such as patient age, availability of matched donors, and the toxicity associated with the treatment, less than half of all patients met the eligibility criteria [17]. The outlook for patients with CML was generally grim.

## Molecular pathogenesis of CML and rationale for TKIs

The association of CML with specific genetic features had been discovered in 1960, when Peter Nowell and David Hungerford identified an aberrant shortened chromosome 22 in cell cultures from patients with CML, referred to now as the Philadelphia (Ph) chromosome after the city where it was discovered [18]. This was the first genetic abnormality to be associated with cancer. The structure of the Ph chromosome was described in detail by Janet Rowley in 1973, noting that the alteration arose from the reciprocal translocation of chromosomes 9 and 22, known as t(9;22) [19]. Early studies in 1984 showed that CML cells expressed an abnormal transcript of the Abelson murine leukemia viral oncogene homolog (*ABL*), suggesting that the gene had been structurally altered in leukemic cells [20, 21]. Subsequent studies demonstrated that CML cells contain a fused transcript of the *ABL* and breakpoint cluster region (*BCR*) genes, providing direct molecular evidence that the reciprocal chromosomal translocation resulted in a fusion product [22]. The identification of the *BCR::ABL1* fusion gene was a pivotal step in understanding the molecular basis of CML.

Although *BCR::ABL1* was found in nearly all patients with CML [18], many researchers were uncertain about its causative role and believed it was simply associated with the disease [23]. However, in 1990, mouse models of CML developed independently in separate laboratories demonstrated the ability of *BCR::ABL1*, as the sole oncogenic abnormality, to cause leukemia [24–27]. A scientific observation had been translated into a clear therapeutic target: the rationale for the development of a BCR::ABL1 inhibitor that could effectively and selectively target leukemic cells had been established.

*BCR::ABL1* encodes a tyrosine kinase located in the cytoplasm which has constitutive activity [28–30]. The BCR::ABL1 kinase continuously activates several signaling pathways such as Ras-mitogen-activated protein kinase (MAPK), phosphatidylinositol 3-kinase (PI3K)/Akt, and signal transducer and activator of transcription 5 (STAT-5), resulting in increased cell proliferation and survival [31]. But BCR::ABL1 does not just kickstart cellular transformation; rather, leukemic cells are dependent on BCR::ABL1 for their survival. *BCR::ABL1* is thus a classic example of oncogene addiction [32].

In 1979, Tony Hunter discovered tyrosine phosphorylation and showed that tyrosine kinases were critical for cell signaling and could become oncogenic when dysregulated [33, 34]. Researchers began focusing on developing drugs capable of blocking kinase activity to disable cancer drivers; however, there was skepticism on whether this could be achieved. Some claimed that no compounds could ever be developed that were specific enough to target one or only a few of the 90 tyrosine kinases present in the human genome without impacting others involved in physiological functions; others maintained that inhibiting a single kinase would not be sufficient to treat a complex disease like cancer [23, 35].

## Evolution of TKIs in CML

### Imatinib (first generation)

#### Preclinical development and early clinical trials

**Fig. 1:**
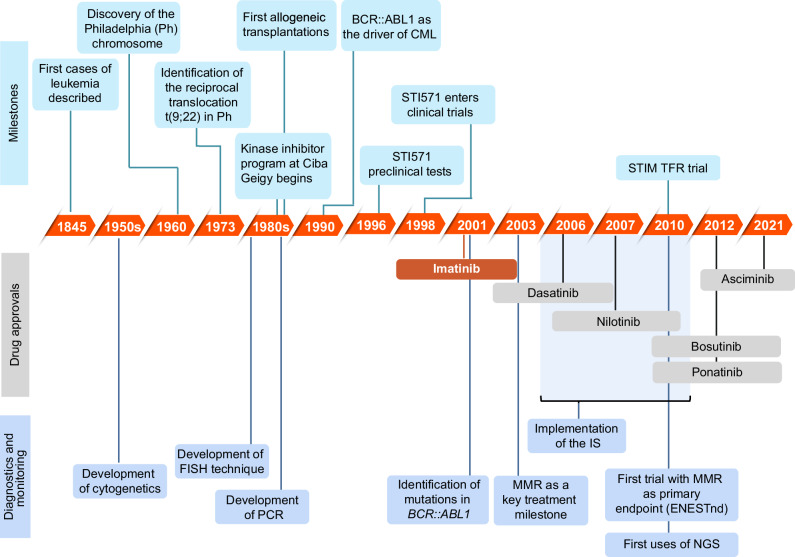
Timeline of key advances in CML therapy over 25 years (1990-present). CML chronic myeloid leukemia, FISH fluorescent in situ hybridization, IS international scale, MMR major molecular response (*BCR::ABL1* on the International Scale [BCR::ABL1^IS^] < 0.1%), NGS next-generation sequencing, PCR polymerase chain reaction, ST571 Signal Transduction Inhibitor 571 (imatinib), STIM Stop Imatinib, TFR treatment-free remission.

In the 1980s, a research team at Ciba-Geigy (now Novartis), under the direction of Nick Lydon and Alex Matter, worked on identifying tyrosine kinase inhibiting compounds for preclinical testing. The team collaborated with oncologist Brian Druker, who believed that CML was the ideal testing ground for kinase inhibitors [23] (Fig. 1).

**Fig. 2:**
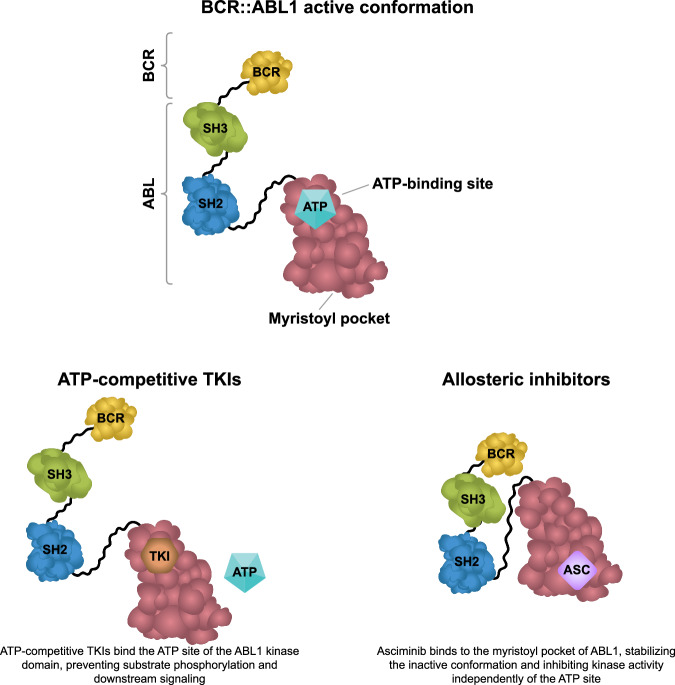
Mechanism of action of BCR::ABL1 TKIs. ABL Abelson kinase, ASC asciminib, ATP adenosine triphosphate, BCR breakpoint cluster region, SH Src homology, TKI tyrosine kinase inhibitor.

A lead compound was identified among thousands in a protein kinase C (PKC) inhibitor screening [36]. The aim was to modify this compound chemically to find the right fit that could bind to BCR::ABL1 and inhibit its activity (Fig. 2) while minimizing interactions with other kinases [23, 36]. From hundreds of novel compounds assessed, a small number was selected for further testing; among these was Signal Transduction Inhibitor 571 (STI571, also known as CGP 57148).

In vitro enzyme inhibition experiments conducted by Druker showed that STI571 potently inhibited ABL1 and BCR::ABL1 without significant effects on other kinases such as epidermal growth factor receptor (EGFR), Src or polyphosphate kinase (PPK); only platelet-derived growth factor receptor (PDGFR) and KIT kinases showed significant inhibition, supporting its selectivity [37, 38]. Accordingly, STI571 killed BCR::ABL1-expressing cells, but not the parental untransfected cells that did not express the oncogene, both in vitro and in vivo. STI571 also inhibited colony formation in samples derived from the bone marrow and blood of patients with CML, but not from patients with other hematological disorders [37].

STI571, or imatinib, as it was later called, is a compound of the 2-phenylaminopyrimidine class. Substitutions at the 6-position of the anilino phenyl ring with a methyl group retained or enhanced activity against tyrosine kinases. The introduction of N-methylpiperazine as a polar side chain greatly improved water solubility and oral bioavailability, which was key to clinical use [36].

Preclinical studies had provided promising results, but bringing imatinib to clinical trials was still challenging; concerns remained about the effectiveness of a drug that aimed to target a single kinase, and about the toxicity of this first-in-class treatment [23]. The first Phase 1 trial started in June 1998, enrolling 83 patients with CML in chronic phase (CML-CP) resistant or intolerant to interferon alpha [39]. Imatinib treatment resulted in complete hematologic response (CHR) in 53 out of 54 patients treated with 300 mg or more, a response rate rarely seen in cancer with a single agent until then; some patients had deeper molecular responses. Furthermore, imatinib was well tolerated: adverse events (AEs) were generally mild, and no dose-limiting toxicities were noted with the wide range of doses assessed [39]. A series of Phase 2 trials in all stages of CML confirmed the efficacy and safety results, and a CHR rate of 96% and survival rate of 76% after 6 years was reported for patients with CML-CP [40–43], a breakthrough for a disease that was until then largely lethal. These results led to the FDA approval in May 2001 of imatinib for the treatment of CML after failure (due to resistance or intolerance) of interferon alpha therapy [44], following a review of less than three months, which was an all-time record [45]. Other countries quickly followed, with the European Medicines Agency approving imatinib in November 2001 [46].

#### IRIS trial and establishment of imatinib as standard of care

The results of the early imatinib clinical trials far exceeded expectations. When the Phase 3 IRIS [International Randomized Study of Interferon and STI571] trial opened in June 2000, it only took 7 months to enroll its target of 1000 patients with newly diagnosed CML-CP [17, 23]. These patients received imatinib 400 mg once daily or interferon alpha plus low-dose cytarabine, which had shown superior efficacy compared to interferon alpha alone [47]. After a median follow-up of 19 months, imatinib showed significantly higher levels of CHR (95.3% vs 55.5% with interferon alpha plus low-dose cytarabine), major cytogenetic response (MCyR [Ph-positive metaphases <35%], 85.2% vs 22.1%) and complete cytogenetic response (CCyR [Ph-positive metaphases 0%], 73.8% vs 8.5%) [17]. It is worth mentioning that MCyR was the primary endpoint for this trial as this was the realistic treatment goal with interferon alpha, and expectations were thus modest for any new agent. Tolerability was good for imatinib, with most AEs being Grade 1 or 2. The extent of imatinib superiority was such that results were disclosed earlier than expected and most patients in the interferon alpha arm were crossed over to receive imatinib after a median of 9 months of therapy in the control arm [23, 48, 49]. A long-term analysis of the IRIS trial with 10.9 years of follow-up reported a 10-year OS rate of 83.3% with imatinib [50]. The 10-year survival rate in the control arm was 78.8%, better than historical data (10-year OS rates <20%); this is likely because of the cross-over to imatinib therapy.

Data from the European Group for Blood and Marrow Transplantation (EBMT) showed that rates of allogeneic HSCT increased from 2000 to 2002 for all indications except two, one being CML; subsequent studies showed that rates of allogeneic HSCT for CML peaked in 1999. Meanwhile, data from the Center for International Blood and Marrow Transplant Research (CIBMTR) showed a 64% decrease in utilization of HSCT in North America from 1998 to 2003 [51–53]. US data collected between 1998 and 2011 showed a 4-fold decrease in the rate of allogeneic HSCT in CML since the introduction of TKIs [54].

## Second-generation TKIs

While most patients with CML-CP had excellent outcomes on imatinib, some patients were refractory to treatment and some had responses that were not durable. Large trials attempted to optimize imatinib therapy, assessing it at higher doses and in combination with interferon alpha and/or low-dose cytarabine [1, 55–57]. Although both approaches resulted in faster and sometimes improved achievement of molecular response, no longer-term benefit compared with imatinib alone was observed.

Thanks to the existing knowledge on the biological mechanisms of disease, studies quickly established that a relevant proportion of patients who relapsed on imatinib had specific mutations in *BCR::ABL1* [58–60], which reduced imatinib’s ability to bind and inhibit tyrosine kinase activity [58, 61, 62]. The T315I (also referred to as a gatekeeper) mutation was the first to be described, conferring a very high level of resistance to imatinib [58]. More than 100 others such as E255V and E255K quickly followed [59, 60]. Although other mechanisms or resistance were also identified (related to BCR::ABL1 overexpression, drug transport, or activation of other signaling pathways), none was as prominent or as clinically actionable as *BCR::ABL1* mutations.

With the proven success of imatinib, pharmaceutical companies sought to develop a second wave of more potent TKIs that could overcome resistance; these became known as second-generation (2G) TKIs. Initial efforts focused on two agents, dasatinib and nilotinib.

Dasatinib is structurally unrelated to imatinib. It is 300-times more potent than imatinib and is active against most tested *BCR::ABL1* mutations that confer resistance to imatinib, the exception being T315I; it has also shown activity against Src kinases [63] and subsequently several other tyrosine kinases including Bruton’s tyrosine kinase (BTK) [64]. The five Phase 2 trials from the START (Src/ABL Tyrosine Kinase Inhibition Activity Research Trials of dasatinib) program confirmed that dasatinib could overcome most imatinib-resistant mutations in the clinic [65–70]. Furthermore, treatment with dasatinib resulted in better outcomes compared with imatinib dose escalation [71]. Later, the Phase 3 trials CA180-034 and CA180-035 [72, 73] showed MCyR in 59% of patients with CML-CP and 39% of patients with CML in accelerated phase treated with dasatinib in second line. Thus, dasatinib was approved for imatinib-resistant/intolerant CML in 2006 [74, 75]. At a daily dose of 100 mg, dasatinib showed early responses in the first-line setting [76] as well as superiority to imatinib in the DASISION trial (12-month CCyR rate 77% vs 66%) [77], which led to its approval for first-line treatment of CML-CP in 2010 [78, 79].

Nilotinib was developed as a structural modification of imatinib to increase its potency against unmutated BCR::ABL1 and to preserve binding to imatinib-resistant BCR::ABL1 mutated forms; it has also shown activity against the discoidin domain receptors (DDR1/2), KIT, PDGFR, and colony stimulating factor receptor-1 (CSF-1R) kinases [80, 81]. It is at least 20-fold more potent than imatinib but, like dasatinib, has no activity against the T315I mutation [63]. In Phase 1/2 clinical trials, nilotinib induced hematological and cytogenetic responses in patients with CML resistant to imatinib [82, 83]. Based on these results, in 2007 nilotinib received approval for CML-CP and CML in accelerated phase in adults resistant to or intolerant of imatinib-based therapy [84, 85]. Nilotinib was also studied as first-line therapy in two Phase 2 studies [86, 87]; the results showed higher CCyR rates vs imatinib. The ENESTnd Phase 3 trial comparing nilotinib 300 mg or 400 mg twice daily vs imatinib in newly diagnosed CML-CP confirmed these results, showing deeper levels of response (major molecular response, MMR; BCR::ABL1 on the International Scale [BCR::ABL1^IS^] < 0.1%) at 12 months for nilotinib (44% at 300 mg twice daily vs 22% for imatinib) [88, 89]. This was the first time MMR had been used as a primary endpoint in a CML clinical trial. In 2010, nilotinib received expanded approval for the treatment of newly diagnosed CML-CP [85, 90].

Bosutinib has a chemical structure that is different from that of imatinib, dasatinib, and nilotinib and closer to the EGFR inhibitor erlotinib; it is also a multi-kinase inhibitor like dasatinib, with activity against Src but not against KIT or PDGFR [91, 92]. Patients with imatinib resistance or intolerance achieved or maintained MCyR rates of 59% after two years of treatment with bosutinib 500 mg once daily [93, 94]. Once again, bosutinib showed activity against several *BCR::ABL1* mutations, but not T315I [91]; importantly, a subgroup analysis showed that bosutinib was also active in patients who were resistant to dasatinib and/or nilotinib as well as imatinib (MCyR rate 32% after a median follow-up of 28.5 months) [95]. The drug was approved by the FDA in 2012 and conditionally by the EMA in 2013 for adults with CML-CP or CML in accelerated or blast phase resistant to prior TKI therapy [96, 97]. In newly diagnosed CML-CP, bosutinib demonstrated superiority to imatinib, with a cumulative MMR rate of 73.9% vs 64.6% by 5 years [98]. Bosutinib was approved for first-line use in CML-CP in 2017 [99].

Other 2G TKIs of interest include radotinib [100, 101], approved in 2012 in South Korea for patients not optimally responding to previous TKIs and in 2015 for newly diagnosed patients, and flumatinib [102, 103], approved in China in 2019 [104].

Although the increased potency of 2G TKIs compared with imatinib was reflected in more patients achieving deeper and faster responses and lower rates of disease progression, none showed improved OS compared with imatinib when used in first line [105, 106]. Furthermore, the higher potency of 2G TKIs, coupled with their potential for inhibition of kinases other than BCR::ABL1, brought along different toxicity profiles.

## Third-generation TKIs

The arsenal against CML now comprised several TKIs, but resistance still proved a challenge. Unlike imatinib and the 2G TKIs that were designed to interact with the ATP-binding site of BCR::ABL1 with high affinity, novel TKIs aimed to specifically target the molecular mechanisms responsible for resistance [107, 108].

Ponatinib, a third-generation TKI, was developed specifically against BCR::ABL1 harboring the T315I mutation, using a computer- and structure-guided strategy [109]. Preclinical studies demonstrated successful targeting of T315I-mutated BCR::ABL1, albeit at higher concentrations than unmutated BCR::ABL1. Ponatinib also inhibited Src and members of the vascular endothelial growth factor receptor (VEGFR), fibroblast growth factor receptor (FGFR), and PDGFR families of tyrosine kinases [109]. In the Ponatinib in Ph+ Acute lymphoblastic leukemia and CML Evaluation (PACE) Phase 2 study, ponatinib showed considerable activity among 449 patients with CML (in chronic, accelerated or blast phase) and Ph+ ALL, regardless of baseline domain mutations and with durable responses [110]. Notably, response rates were higher among patients with CML-CP harboring the T315I mutation compared with all patients with CML-CP (by 12 months: MCyR: 70% vs 56%; CCyR: 66% vs 46%; MMR: 56% vs 34%) [110]. Ponatinib was approved in 2012 for CML-CP or CML in accelerated or blast phase resistant or intolerant to prior TKI therapy; however, cardiovascular safety concerns (in particular, arterial occlusive events) led to a temporary suspension of the drug. In early 2014 ponatinib was re-licensed exclusively for adult T315I-positive CML or CML resistant to multiple TKIs in all phases [111, 112]. To address the safety concerns, the OPTIC trial evaluated the efficacy and safety of ponatinib using a response-based, dose-reduction strategy in patients with CML-CP resistant to ≥2 TKIs or harboring the T315I mutation. The starting dose of ponatinib was 45 mg, 30 mg, or 15 mg daily; patients in the 45 mg and 30 mg cohorts had their dose reduced to 15 mg daily upon achieving BCR::ABL1^IS^ ≤ 1% [113]. This strategy resulted in lower rates of arterial occlusive events, fewer dose reductions related to AEs and longer median time on therapy compared with the initial fixed-dose strategy followed in PACE, leading to higher rates of OS and progression-free survival (PFS) [114]. These results led to approval of a new indication in 2022, for response-based dosing in CML-CP resistant or intolerant to ≥2 TKIs or with T315I mutation, as well as CML in accelerated phase, CML in blast phase and Ph+ ALL [112].

Olverembatinib was developed using computational structural modeling to target T315I-mutated BCR::ABL1 [115]. Of note, preclinical studies showed that olverembatinib was active against multiple *BCR::ABL1* mutations, including T315I and compound mutations against which ponatinib was not effective [115, 116]. As a multikinase inhibitor, olverembatinib also decreased the activity of FLT3, FGFR, KIT and other oncogenic kinases [117]. A Phase 2 study showed 3-year cumulative rates of CCyR and MMR of 69% and 56%, respectively, in patients with CML-CP, and 47% and 45%, respectively, in CML in accelerated phase [118]. In a more recent study of olverembatinib in patients with relapsed/refractory CML-CP, the CCyR and MMR rates were comparable between patients with or without T315I mutation; importantly, olverembatinib was active in patients with ponatinib resistance [119]. Although cardiovascular events were reported on treatment, the rates were lower than those observed with ponatinib, albeit in a younger patient population [118]. In November 2021, olverembatinib was approved in China for the treatment of TKI-resistant CML-CP and CML in accelerated phase with T315I mutation, and in November 2023, approval was expanded for the treatment of CML-CP resistant or intolerant to first- and second-generation TKIs [120]. Registrational studies in other countries are ongoing.

The third-generation novel TKI vodobatinib has shown promising results in a Phase 1/2 trial in patients with CML-CP, CML in accelerated phase, or CML in blast phase treated with at least three previous TKIs, including ponatinib and asciminib [121, 122]. Unlike other third-generation TKIs, vodobatinib is not effective against the T315I mutation.

## Novel agents with innovative mechanisms of action

Third-generation TKIs were more potent than previous drugs and had activity against resistance-conferring mutations, particularly T315I. However, there was concern about their use due to cardiovascular toxicity. Similar concerns also existed for 2G TKIs, particularly when used in older patients or those with cardiovascular comorbidities. ATP-competitive TKIs developed so far targeted other kinases besides BCR::ABL1, which could account for the off-target toxicity [123]. New agents were needed with improved safety profiles and higher specificity for BCR::ABL1.

The ABL1 and ABL2 proteins carry an N-terminal glycine residue which is post-translationally modified to be myristoylated. This residue is involved in regulating kinase activity by binding to a pocket within ABL1, inducing an inactive state [124, 125]. This self-regulating mechanism in ABL1 is lost in BCR::ABL1 as the BCR fragment replaces the region of ABL1 containing the myristoylation site [124].

Asciminib, the first BCR::ABL1 inhibitor that works by Specifically Targeting the ABL Myristoyl Pocket (STAMP), was developed to bind to the myristoyl pocket of ABL, a unique region present in ABL1/2 kinases and their fusion oncoproteins including BCR::ABL1 [126]. Asciminib binding results in BCR::ABL1 reverting to an inactive form, thereby inhibiting kinase activity in an allosteric manner [127]. With a different mechanism of action to ATP-competitive TKIs, asciminib retains activity against *BCR::ABL1* mutations in the ATP-binding site, including T315I, which was confirmed in animal studies with nilotinib-resistant tumors [128]. In the first-in-human Phase 1 study, asciminib monotherapy achieved an MMR rate of 48% at 12 months in patients with CML-CP without the T315I mutation who had received ≥2 prior TKIs [129]. Among patients with the T315I mutation, 24% achieved MMR by 12 months. Asciminib was well tolerated in this heavily pretreated patient population, with most non-hematological AEs being Grade 1 or 2 [129]. These results were confirmed in the pivotal ASCEMBL Phase 3 study, assessing asciminib 40 mg twice daily vs bosutinib 500 mg daily in 233 patients with CML-CP previously treated with ≥2 TKIs [130]. The MMR rate at week 24 was 25.5% with asciminib and 13.2% with bosutinib; at week 96, the rates were 37.6% vs 15.8%, respectively [131]. Asciminib was also associated with lower rates of Grade ≥3 AEs and AEs leading to discontinuation. In 2021, asciminib was approved in adult Ph+ CML-CP previously treated with ≥2 TKIs and CML with the T315I mutation [132, 133]. The efficacy of asciminib in newly diagnosed Ph+ CML-CP was evaluated in the ASC4FIRST trial, comparing asciminib 80 mg once daily with all TKIs approved for first-line treatment (imatinib, dasatinib, nilotinib, and bosutinib) as selected by investigators [134, 135]. Overall, the MMR rates were 67.7% vs 49.0% at 48 weeks and 74.1% vs 52.0% at 96 weeks, respectively, with better tolerability for asciminib vs investigator-selected TKIs, including fewer dose modifications, fewer Grade ≥3 AEs, and a lower risk of treatment discontinuation due to AEs [134]. Based on these results, asciminib received approval for adult Ph+ CML-CP, including first-line use in 2025 [136, 137].

Two new BCR::ABL1 inhibitors that work by STAMP, TERN-701, and TGRX-678 are currently undergoing Phase 1 or Phase 2 trials, with promising early results [138, 139].

## Use of TKIs in combination

The currently available TKIs have two different mechanisms of action, which target separate binding sites within BCR::ABL1 (Fig. 2). Early results with asciminib showed that this drug could be used in combination with ATP-competitive TKIs to address or prevent the development of resistance [128, 129, 140]. Addition of asciminib to imatinib resulted in faster and deeper responses than continuing imatinib or switching to nilotinib in the Phase 2 ASC4MORE study [141]. Following on these results, the Phase 2 FASCINATION trial aimed to assess asciminib in combination with imatinib, dasatinib, or nilotinib in newly diagnosed patients with CML-CP [142]. High rates of deep molecular response (DMR; MR^4^ or deeper) with combination treatment were associated with decreased tolerability, with 17% of patients discontinuing treatment by one year, mostly due to AEs. However, Grade ≥3 AE rates decreased from 37.6% at one year to 9.6% at 3 years [143]. Longer-term data confirmed the high levels of DMR at 3 years (MR^4^, 65%; MR^4.5^, 45%; MR^5^, 27%; MR^5.5^, 20%) and showed activity in patients harboring mutations in *ASXL1*, which are associated with poor prognosis.

A combination of asciminib and ponatinib has been proposed to be tolerable and effective in advanced CML or Ph+ ALL [144, 145], and further studies are needed to assess its safety and efficacy in these settings. Treatment with TKIs in combination remains investigational as the benefit of potentially increased efficacy is counterbalanced by decrease in tolerability as the result of two combined AE profiles.

Ultimately, successive generations of TKIs were enabled by shared clinical trial infrastructures, harmonized endpoints, and global trial programs enrolling patients across multiple sites worldwide. These programs accelerated regulatory approval and adoption into practice globally, highlighting the impact of collaboration across countries and continents.

Overall, TKIs dramatically improved survival rates for patients with CML, from 10–20% to rates close to those of the general population for those patients who responded to treatment [11, 146], with a considerably improved side effect profile compared to previous therapies (Table 1 and Table 2). A new score to assess risk (the European Treatment and Outcome Study [EUTOS] long-term survival or ELTS score) was developed that took into account that the cause of death of most patients was no longer CML [147]. Prolonging survival had been the original treatment goal for pre-TKI therapies, but now CML had been transformed into a chronic disease with low mortality rates. Furthermore, TKIs had proven so effective that new techniques had to be developed to detect ever lower levels of residual disease. TKIs did not only revolutionize CML therapy; they revolutionized patient management as well.

**Table 1:** Comparison of approved TKIs for CML-CP.

TKI	Key trials	Number of patients with CML-CP	Setting	Treatment	Response rate
**Pretreated**					
Imatinib	[40]	532	Previously treated	Imatinib 400 mg QD	**CCyR by 18 months:** 41%
Dasatinib	CA180-034 [72]	670	Resistant or intolerant to imatinib	Dasatinib 100 mg QD vs 50 mg BID vs 140 mg QD vs 70 mg BID	**CCyR by 6 months:** 41% vs 42% vs 44% vs 45%
Nilotinib	[82]	280	Resistant or intolerant to imatinib	Nilotinib 400 mg BID	**CCyR at 6 months:** 31%
Bosutinib	[93]	288	Resistant or intolerant to imatinib	Bosutinib 500 mg QD	**CCyR at 6 months:** 23% **CCyR by 2 years:** 41%
Ponatinib	PACE [295]	267 (of whom 64 with T315I)	Resistant or intolerant to dasatinib or nilotinib, or with T315I	Ponatinib 45 mg QD	**CCyR by 1 year:** 46% (66% in patients with T315I)
Asciminib	ASCEMBL [130]	233	Pretreated with two or more TKIs	Asciminib 40 mg BID vs bosutinib 500 mg QD	**CCyR at 6 months:** 40.8% vs 24.2% **MMR at 6 months:** 25.5% vs 13.2%
Olverembatinib	[119]	62	Resistant or intolerant to at least two TKIs	Olverembatinib 30 mg, 40 mg or 50 mg QOD in 28-day cycles	**CCyR:** 60.8% **MMR:** 42.4%
**Newly diagnosed**					
Imatinib	IRIS [17]	1106	Newly diagnosed	Imatinib 400 mg QD vs interferon alfa plus low-dose cytarabine	**CCyR at 18 months:** 76.2% vs 14.5%
Dasatinib	DASISION [77]	519	Newly diagnosed	Dasatinib 100 mg QD vs imatinib 400 mg QD	**CCyR by 1 year:** 77% vs 66% **MMR by 1 year:** 46% vs 28%
Nilotinib	ENESTnd [88]	846	Newly diagnosed	Nilotinib 300 mg BID vs nilotinib 400 mg BID vs imatinib 400 mg QD	**CCyR by 1 year:** 80% vs 78% vs 65% **MMR at 1 year:** 44% vs 43% vs 22%
Bosutinib	BFORE [296]	536	Newly diagnosed	Bosutinib 400 mg QD vs imatinib 400 mg QD	**CCyR by 1 year:** 77.2% vs 66.4% **MMR at 1 year:** 47.2% vs 36.9%
Radotinib	RERISE [101]	241	Newly diagnosed	Radotinib 300 mg BID vs radotinib 400 mg BID vs imatinib 400 mg QD	**CCyR by 1 year:** 91% vs 82% vs 77% **MMR by 1 year:** 52% vs 46% vs 30%
Asciminib	ASC4FIRST [134]	405	Newly diagnosed	Asciminib 80 mg QD vs investigator-selected TKIs (imatinib 400 mg QD / dasatinib 100 mg QD / nilotinib 300 mg BID / bosutinib 400 mg QD)	**MMR at 1 year:** 67.7% vs 49.0%

*BID* twice daily, *CCyR* complete cytogenetic response, *CML-CP* chronic myeloid leukemia in chronic phase, *MMR* major molecular response, *QD* once daily, *QOD* every other day.

**Table 2:** Safety profile of approved TKIs in CML.

Adverse Events	Imatinib [17, 39, 50]	Dasatinib [76, 77]	Nilotinib [88, 89]	Bosutinib [98, 296]	Ponatinib [113, 295]	Asciminib [129, 134, 135]
**Most common events**^**a**^	Edema, muscle cramps, joint pain, nausea, diarrhea	Cytopenias, headache, fatigue, diarrhea, increased liver enzymes	Cytopenias, rash, increased liver enzymes, hyperglycemia, hypercholesterolemia	Diarrhea, nausea, increased liver enzymes, cytopenias	Arterial occlusion, rash, dry skin, abdominal pain, cytopenias	Fatigue, rash, cytopenias, vomiting
**Distinctive risks**^**a**^	Fluid retention, mild cytopenias	Pleural effusion, pulmonary arterial hypertension	Arterial occlusive events, hyperglycemia, hyperlipidemia, QT prolongation	Hepatotoxicity, pronounced GI toxicity, renal impairment	High arterial/vascular thrombosis risk, hypertension, pancreatitis	Hypertension, lipase elevation, mild cytopenias

^a^Most common events and distinctive risks are presented irrespective of causality.

## Monitoring response and defining treatment goals in the TKI era

### Diagnosis and monitoring

Originally, the structure of the t(9;22) translocation was established by Giemsa or G-banding, a technique that stains chromosomes to facilitate their identification [19]. This technique was subsequently used to diagnose CML, although it was labor intensive and required culturing of bone marrow cells; it remains a recommended test at diagnosis to this date. Later, fluorescence in situ hybridization (FISH) facilitated the process and increased sensitivity of detection [148]. The advent of PCR [149] greatly improved CML diagnosis. Given its disease-specificity, *BCR::ABL1* was an ideal target for amplification, and soon specific RT-PCR methods were developed for detection [150].

The detection of *BCR::ABL1* had other uses than simple diagnosis. Before imatinib, therapeutic efficacy was assessed mainly by determining hematologic or cytogenetic responses, which provided prognostic clues. Patients who achieved CCyR had better outcomes in terms of quality and duration of response, as well as OS [151–153]. Despite this, even patients with undetectable residual disease as determined by cytogenetic analysis (G-banding or FISH) could still harbor small numbers of leukemic cells that could later induce disease relapse [148, 154]. Furthermore, imatinib induced deeper responses that could not be adequately measured by the traditional methods available at the time, prompting the need for more sensitive and standardized residual disease detection. As a result, quantitative PCR–based assays became necessary to detect very low levels of *BCR::ABL1* transcripts [148, 155].

The first quantitative PCR methods were used to detect residual disease post-HSCT and proved extremely laborious. Accurate and sensitive quantitative RT-PCR (qRT-PCR) techniques were developed that were far simpler and could stratify patients further, identifying those with deeper levels of response and predicting risk of disease relapse [15, 156–161].

Thanks to these methods, the Phase 3 IRIS study comparing imatinib with interferon alpha and low-dose cytarabine not only established imatinib as the standard of care in CML: it also determined the prognostic value of molecular response by measuring the levels of blood *BCR::ABL1* transcripts in patients who achieved CCyR, which was an exploratory biomarker investigated in the study. The amount of residual disease at 12 months, measured by qRT-PCR in terms of log reduction of *BCR::ABL1* transcripts, could predict PFS and the risk of disease progression for patients with CML achieving CCyR [162]. Specifically, imatinib-treated patients who had a reduction of at least three log in the level of *BCR::ABL1* transcripts in peripheral blood (relative to a standardized baseline from pretreated samples) had negligible risk of disease progression over the subsequent 24 months [162]. These results were confirmed by a later analysis, showing that patients who achieved a greater than three-log reduction in *BCR::ABL1* levels by 18 months had extremely durable responses and very low risk of disease progression, leading to this level of response being termed major or MMR [163]. Since the degree of reduction in *BCR::ABL1* levels in response to therapy was associated with long-term outcomes (albeit not with longer OS beyond that achieved with CCyR), it became clear that optimal treatment was based on appropriate monitoring of response to therapy. As such, the results of qRT-PCR became the basis for routine therapeutic decisions in CML [6]. Monitoring of *BCR::ABL1* levels is recommended at least every three months until confirmed MMR is achieved; intervals can then increase to 6 months, although monitoring should remain frequent in patients considering treatment discontinuation [164–167].

Monitoring in the IRIS study involved laboratories based in Adelaide, London, and Seattle. Investigators had understood that inter-laboratory variability could pose a challenge in terms of comparing results across studies and countries; they therefore expressed the levels of *BCR::ABL1* as a percentage normalized to the expression of a control gene (in this case, the *BCR* gene) [162]. This compensated for variability in mRNA quality and efficiency of the qRT-PCR reaction. However, differences in *BCR::ABL1* values were still detected across the three laboratories. A set of 30 baseline samples was then selected as internal reference, with each laboratory measuring *BCR::ABL1* levels in this set and using it as a standardized baseline to normalize their results; *BCR::ABL1* levels were then expressed as a percentage of this reference baseline rather than the corresponding baseline value for each patient. This approach substantially improved comparability of results across laboratories and prompted a redefinition of response levels: MMR, for example, was defined as a three-log reduction in *BCR::ABL1* levels from the IRIS standardized baseline [162]. CCyR was considered to be roughly equivalent to a two-log reduction (MR^2^) [168].

Calls and recommendations for harmonization of *BCR::ABL1* levels across laboratories and countries followed, with laboratories worldwide coming together to find a way to standardize results across countries [169]. The Adelaide laboratory established a system where other laboratories could send samples for testing, allowing for the calculation of a laboratory-specific conversion factor; this helped the laboratories to convert their results to a reference scale that was shared by all. The EUTOS collaboration also sought to standardize national reference laboratories for quantitative results and assessment of DMR [170–172]. The process culminated in the implementation of the International Scale (IS), which is essentially the same as the IRIS standardized baseline, to be used with *ABL1*, *BCR*, or *GUSB* as control genes [163, 169, 173, 174]. In 2010, the WHO approved the first International Genetic Reference Panel for quantitation of *BCR::ABL1* mRNA as an accredited standard for IS calibration [175]. Although not the first attempt at standardization of cancer results across the world, the IS was likely the first successful, globally accepted standardization of molecular response in a specific cancer.

Novel monitoring techniques are emerging in CML. Digital PCR (dPCR) is a highly sensitive and accurate method; results show that dPCR could provide improved precision and sensitivity compared with qRT-PCR, particularly for deep responses [176, 177], highlighting its potential as the standard for disease monitoring in CML.

*BCR::ABL1* mutations associated with resistance to treatment can be identified by DNA sequencing, and direct sequencing methods such as the Sanger method were traditionally used for this purpose. However, these methods have limited sensitivity, being unable to detect mutations present in <20% of Ph+ cells [164, 178]. Since the presence of mutations even at low levels is important for clinical decisions, new methods such as next-generation sequencing (NGS) are being explored in CML [179]. NGS can detect point mutations in *BCR::ABL1* with a sensitivity of 1% and, unlike Sanger sequencing, can discriminate between polyclonal (different mutations in different leukemic clones) and compound mutations (different mutations in a single *BCR::ABL1* transcript). Moreover, NGS has detected additional low-level mutations in a substantial proportion of patients who had mutations identified by Sanger sequencing [180]. The potential for NGS to improve clinical decision-making in patients with TKI resistance led to this technique being included in clinical recommendations [165–167]; however, it is expensive and not yet standardized.

Advances in diagnostic and monitoring of CML have gone hand in hand with the development of new therapies. In a similar way, treatment milestones and goals have also changed as more effective therapies became available.

## Evolution of treatment goals in CML

As CML therapies became more effective, so the treatment goals evolved. Before TKIs, the main aim of CML treatment was to improve survival [105]; only HSCT harbored the possibility of a cure. Once it became clear that TKIs dramatically improved OS, clinicians searched for levels of response that could predict improved survival and identified a clear association between CCyR and OS [151, 152]. The IRIS trial also showed that, among patients who had achieved CCyR, those in MMR had higher rates of PFS [162]. MMR thus became a key goal of therapy and the primary endpoint of TKI trials. [166, 167].

Both the European LeukemiaNet (ELN) treatment recommendations and the National Comprehensive Cancer Network® (NCCN®) Clinical Practice Guidelines in Oncology (NCCN Guidelines®) define treatment success through standardized, time‑dependent molecular response milestones assessed by qRT-PCR on the IS [166, 167, 181]. The ELN recommendations include minimum *ABL1* and *GUSB* control transcript numbers in case of negative results to ensure accuracy and sensitivity of qRT-PCR results [166]. Early molecular response (defined as BCR::ABL1^IS^ ≤ 10% at 3 months) is proposed as a prognostic landmark in both sets of recommendations, with achievement associated with favorable long‑term outcomes [166, 167]. By 12 months, CCyR is the main treatment goal, and suboptimal response leads to reassessment of treatment strategy. Collectively, the ELN recommendations and NCCN Guidelines^®^ reinforce a milestone‑driven monitoring and treatment strategy that integrates evolving long‑term goals. The recent 2025 ELN recommendations also emphasize flexibility in treatment decisions based on individualized approaches for each patient [166].

When TKIs achieved near-normal survival, the goals of CML treatment shifted towards a patient-centric focus; improving QoL, particularly taking into account the long-term nature of treatment, became a main aim of therapy.

TKIs have a much more favorable toxicity profile compared to previous CML therapies; however, all TKIs are associated with side effects that have a negative impact on QoL [182]. Given that these drugs are taken for a long period of time, even low-grade side effects may become an issue [182, 183]. In the CML SUN survey, a substantial proportion of patients expressed that the disease and its treatment impacted their physical and emotional health, and also every aspect of their lives, including their mental health, work, studies, and social life [184]. Expectations of treatment may also change over time, with patients at treatment start prioritizing minimal side effects and low impact on their work life, and those at later stages shifting their priorities towards tolerability and resilience [185]. The cost of long-term TKI therapy, sometimes lifelong, was also initially of concern, but is now a lesser consideration thanks to generic forms of imatinib and dasatinib resulting in much lower overall costs.

The emergence of several successful TKIs over the past 25 years provided clinicians with a choice in terms of selecting treatment [166, 167]. This has ushered in a more personalized type of treatment for CML, where clinicians can take into account factors such as patient age, aims of therapy (survival, TFR), comorbidities, risk score, and financial situation in order to tailor therapy to each patient and select a treatment that also maximizes QoL.

A more recent goal of treatment is the achievement of not just MMR, but deeper levels of response (4 and 4.5 log reductions in BCR::ABL1^IS^, also termed MR^4^ and MR^4.5^). As monitoring techniques improved in parallel with developments in therapy, it was noted that the depth of response increased with time for patients on TKIs, with *BCR::ABL1* levels becoming practically undetectable in some patients [186, 187]. This led clinicians to think that molecular remission might indicate successful eradication of leukemic cells in some patients, and thus, the possibility of cure was raised.

## Treatment-free remission

The concept of TFR in CML first emerged in 2002, when 15 patients who had achieved CCyR stopped treatment with interferon alpha; after a median follow-up of 36 months, 7 patients (47%) had not experienced relapse [188]. For most clinicians, the existence of residual disease discouraged treatment interruption; however, a small proportion of patients treated with imatinib in the frontline setting were shown to have undetectable *BCR::ABL1* levels [162], raising the possibility of successful treatment cessation. A small pilot study with 12 patients with undetectable residual disease for more than two years found that 50% of patients maintained undetectable *BCR::ABL1* levels for 18 months after imatinib interruption [189]; for those who relapsed, *BCR::ABL1* transcripts were detected early (within 6 months of treatment interruption) and patients regained response once retreated with imatinib. This led to the Stop Imatinib (STIM) 1 study, where imatinib was discontinued in 100 patients with undetectable residual disease by qRT-PCR after two or more years of treatment [5]. Overall, 39% of patients maintained undetectable disease after discontinuation of imatinib. Once again, most relapses occurred within 6 months and all patients who relapsed responded to retreatment with imatinib. The TWISTER study, which was conducted in parallel, reported similar results, with 47.1% of patients with undetectable *BCR::ABL1* mRNA able to maintain response off therapy [190, 191]. The STOP 2G-TKI trial reported higher rates of TFR success in patients treated with dasatinib and nilotinib (63% at 12 months) [192]. Notably, the success rates were lower for patients with previous resistance to imatinib compared with patients on frontline 2G TKIs or with previous intolerance to imatinib. Later, the EURO-SKI, DASFREE, ENESTop and ENESTfreedom studies confirmed successful discontinuation of dasatinib and nilotinib [193–196]. A list of selected TFR trials is presented in Table 3.

**Table 3:** Landmark TFR trials in CML.

Trial name	Number of patients	Treatment	Criteria for discontinuation	Rates of TFR success
STIM1 [5]	100	Imatinib	• Chronic or accelerated phase • Imatinib treatment ≥3 years • CMR^a^ ≥2 years	41% at 12 months
TWISTER [191]	40	Imatinib	• CML-CP • Imatinib treatment ≥3 years • UMRD^b^ ≥ 2 years	47.1% at 24 months
EURO SKI [193]	755	Any TKI (predominantly imatinib)	• CML-CP • TKI therapy ≥3 years without failure^c^ • Sustained MR^4^ ≥ 1 year	50% at 24 months
ENESTfreedom [196, 297]	190	Frontline nilotinib	• CML-CP • In MR^4.5^ after ≥2 years nilotinib • Sustained DMR^d^ for 1 year of nilotinib consolidation	51.6% at 48 weeks and 42.6% at 5 years
ENESTop [195, 298]	126	Nilotinib after frontline imatinib	• CML-CP • ≥3 years of treatment (> 4 weeks with imatinib, then ≥2 years with nilotinib) and in MR^4.5^ on nilotinib • No confirmed loss of MR^4.5 e^ for 1 year of nilotinib consolidation	58% at 48 weeks and 42.9% at 5 years
DASFREE [194]	84	Dasatinib (first or subsequent line)	• CML-CP • Dasatinib ≥2 years and in MR^4.5^ on dasatinib ≥1 year	46% at 2 years
MD Anderson Cancer Center [213]	284	Imatinib, 2G TKIs and ponatinib (first, second line or beyond)	• CML-CP • Achieved DMR on TKI • Median duration of DMR 74 months	79% at 5 years

*2G* second generation, *CMR* complete molecular remission, *DMR* deep molecular response, *ELN* European LeukemiaNet, *qtRT-PCR* quantitative real-world polymerase chain reaction, *UMRD* undetectable minimal residual disease.

^a^CMR was defined as remission lasting more than two consecutive years and confirmed with five instances of *BCR::ABL1* analysis by qRT-PCR over these 2 years.

^b^UMRD was defined as no detectable *BCR::ABL1* mRNA in any peripheral blood or bone marrow sample tested for the past 2 years

^c^According to ELN 2013 criteria [164].

^d^Sustained DMR was defined as MR^4.5^ in the last assessment, no assessment worse than MR^4^ and ≤2 assessments between MR^4^ and MR^4.5^.

^e^Confirmed loss of MR^4.5^ was defined as *BCR::ABL1*^IS^ > 0.0032%, confirmed in a second assessment within 4 weeks.

These studies showed that interruption of TKI treatment could be safe and proposed TFR as a valuable new goal of CML treatment, a molecular cure that would allow patients to reduce the burden of side effects from long-term medication. In the 2020 ELN recommendations, TFR was included as one of the goals of CML treatment, with discontinuation to be considered in patients with durable DMR [165].

An unexpected finding in TFR studies was the emergence of TKI withdrawal syndrome. A substantial proportion of patients experienced musculoskeletal pain upon TKI cessation, which improved slowly over time; the effect appeared to be class related as it was observed with all discontinued TKIs [197–199]. This syndrome can respond to steroids and anti-inflammatory medications [197] and is mostly low grade and self-limiting. Imatinib withdrawal syndrome has been associated with better outcomes post-discontinuation [200].

Not all patients are eligible to attempt TFR; clinical and disease factors are important predictors of TFR success. Depth of response is a precondition for TFR, with studies recruiting patients in MR^4^ or MR^4.5^; however, undetectable disease by digital droplet (ddPCR) is associated with higher chances of TFR success compared with undetectable disease by qRT-PCR [198, 201]. The duration of treatment and duration of DMR also correlate with TFR success across TKIs [193–195, 202].

Current recommendations for TFR eligibility include CML-CP (no accelerated phase or blast phase), duration of TKI therapy of 3–5 years, and duration of DMR > 2 years [166, 167]. Most studies have confirmed that relapses occur most commonly within the first 6 months of stopping treatment. The risk of relapse is very small for patients who remain in DMR for 2 or 3 years after treatment cessation [203, 204]; however, frequent monitoring is still recommended for all patients in TFR [166]. Some patients who relapse on TFR and achieve DMR again on retreatment can successfully attempt a second TFR phase [166, 205–207]. Although TFR is generally considered safe, cases of blast phase following treatment cessation have been reported [208–211].

The current overall success rate for TFR is approximately 25%, taking into account rates of eligibility and success, which in most studies are about 50% each [212]. In the MD Anderson trials, the estimated TFR success rate was approximately 80% [213]; however, these studies continued TKI therapy after a DMR (MR^4^ or deeper) duration of 5 years or more rather than 1 or 2 years as in other trials. Given the correlation between depth and length of response and TFR eligibility, DMR has become an emerging treatment goal that goes hand in hand with TFR. Because a greater percentage of patients achieved DMR with 2G TKIs compared with imatinib, it is possible that a larger proportion of patients on these agents may be eligible for TFR, and currently treatment selection takes into account whether TFR is a goal of treatment [50].

## Global access and health economics

### Cost-effectiveness of TKIs

TKIs transformed CML outcomes, but their success revealed a persistent economic barrier: lifelong therapy is expensive, and affordability remained a major challenge despite excellent clinical results [12, 214]. The subsequent patent expiry and generic imatinib entry into the market sharply reduced prices, reported as up to 98% lower than branded TKIs; this improved cost-effectiveness and feasibility of access in low- and middle-income countries (LMICs) [215–217]. In parallel, assistance programs helped turn clinical evidence into real-world survival: since 2002, initiatives such as GIPAP (Novartis) and CMLPath to Care (The Max Foundation, Seattle, WA, USA) have provided free imatinib and other TKIs to >90,000 patients across 75 LMICs [218, 219]. National policy tools accelerated this shift: in India and Brazil, tiered pricing and compulsory licensing enabled local TKI production at a fraction of originator costs [220–222]. Meanwhile, health technology assessment and reimbursement frameworks across Europe, Asia, and Latin America increasingly applied value-based pricing and cost-effectiveness thresholds, accelerating public access to TKIs. In this context, affordable generic imatinib is now widely accessible globally and may be at least 350-fold cheaper than dasatinib, nilotinib, or bosutinib [12]. Generic forms of other TKIs are also widely available.

## Remaining challenges and future directions

Key remaining challenges in CML include the persistently poor prognosis of blast phase disease (median survival ~1 year) and the lack of an established optimal regimen; ongoing studies with TKI plus chemotherapy combinations, dual TKI strategies, and emerging immunotherapies are trying to address this [223, 224]. In addition, primary and acquired TKI resistance mediated by *BCR::ABL1* mutations and BCR::ABL1-independent mechanisms such as drug efflux, alternative signaling, and additional chromosomal alterations (e.g., mutations in genes such as *ASXL1*) limit positive outcomes and lack clear evidence-based management [225–231]. Eradication of TKI-insensitive leukemic stem cells also remains unresolved and will likely require targeting non-BCR::ABL1 pathways [232–237]. Finally, TFR has shifted expectations toward cure, but sustained TFR is achievable in only a minority of patients, highlighting the need for better predictors, broader eligibility, and more potent first-line approaches, as well as deeper understanding of immune determinants of successful discontinuation [238–241].

### Novel TKIs and other CML strategies

Novel TKIs are currently under development, which aim for greater selectivity to minimize off-target toxicity and address resistance challenges [121, 122, 138, 139]. The novel third-generation TKI ELVN-001 is highly selective in terms of kinase inhibition and has shown activity against the T315I mutation in preclinical studies; it is currently in Phase 1 trials [242, 243].

Aside from TKIs, brand new treatment approaches are being tested in CML. Preclinical studies have identified novel agents that inhibit BCR::ABL1 kinase activity through blocking protein-protein interactions: two main ones are monobodies and dimerization inhibitors. Monobodies are non-immunoglobulin binding proteins based on a human fibronectin III domain as a molecular scaffold; unlike antibodies or antibody fragments, they do not contain a disulfide bond, which makes them suitable for targeting intracellular molecules [244, 245]. These agents can interfere with kinase activity by blocking the necessary interaction between BCR::ABL1 Src homology 2 (SH2) and kinase domains and impeding phosphorylation. Similarly, dimerization inhibitors are peptides that imitate the dimerization domain of BCR::ABL1, also interfering with phosphorylation and activity [246]. Both approaches have shown encouraging results in vitro.

In addition to kinase activity, BCR::ABL1 has scaffolding functions that have been shown to contribute to leukemogenesis [247–250]; completely eliminating BCR::ABL1 may then elicit a more powerful anti-leukemic effect compared to inhibition of kinase activity alone, even if the latter is total. Proteolysis-targeting chimeras (PROTACs) are an exciting new type of therapy that can fully eliminate BCR::ABL1 from leukemic cells, abrogating both kinase activity and scaffolding functions [250]. PROTACs take advantage of ubiquitination, a process that targets proteins for degradation [251]. They consist of a ‘warhead’, which actively binds to the protein target, connected via a linker section to an E3 ligase recruiter, part of the ubiquitination system. The purpose of the warhead is to bring the target protein into close proximity with the E3 ligase, which then ubiquitinates the target, marking it for degradation [250]. All approved TKIs except nilotinib have been employed successfully as warheads in BCR::ABL1-targeting PROTACs. To date, these compounds have only been tested in preclinical studies.

Immune-stimulating approaches have shown success in several cancer types; given the evidence suggesting a key role of the immune system in maintaining response during TFR, interest has grown in terms of immunomodulating approaches in CML (reviewed in [252]). Strategies include interferon alpha in combination with TKIs, chimeric antigen receptor (CAR)-T cell therapy, and use of targeted immunotherapies such as blinatumomab to be used in patients with resistance to several TKIs and those with CML in blast phase. On the other hand, checkpoint inhibitors are currently being assessed in CML alone or in combination with chemotherapy (reviewed in [253]) or in combination with TKIs (reviewed in [254]).

## Lessons learned for other malignancies

CML holds a special place in oncology because it was the first ever example of a cancer driven by one main targetable mutation. This created a model now used across many cancer types to find the defining alteration, design a targeted drug, and follow response with molecular tests over time [23, 255].

CML taught three broad lessons for precision oncology. First, if a cancer depends mainly on one signaling pathway, shutting that pathway down can induce deep and lasting benefit [17, 39, 106]. Second, outcomes of targeted therapy are best when response is tracked closely. Routine qRT-PCR monitoring turned follow-up into a quantitative, real-time guide for treatment decisions [106, 174, 256]. Third, resistance often follows a biological pattern, as new kinase mutations can reactivate signaling. Therefore, understanding the biology of disease helped create next-generation drugs and guide treatment sequencing [58, 106, 257].

The revolutionary advances in CML forced clinicians to rethink cancer treatment, managing it as a chronic disease and aiming to maintain QoL during long-term treatment [17, 50]. This pushed oncology towards a chronic-care model that includes regular long-term monitoring and supporting adherence and patient preferences over time [166, 167]. In this model, CML has also reshaped the meaning of cure for the proportion of patients that achieve successful TFR [5, 238].

### CML as a model for precision oncology: alternative indications for BCR::ABL1 TKIs

Once the success of imatinib in CML was established, the search widened across malignancies (including solid tumors) where an activated kinase that can be targeted by imatinib is an essential driver and a clear target for therapy [39, 106, 258–261].

**Ph+ acute lymphoblastic leukemia (ALL):** An obvious target for BCR::ABL1 TKIs is Ph+ ALL, which has the same driver as CML; the development of TKIs for the treatment of Ph+ ALL occurred largely in parallel with CML. Indeed, imatinib was first tested in patients with Ph+ ALL as early as 1999 [262] and trials assessing imatinib in combination with chemotherapy in Ph+ ALL soon followed [263, 264]. TKIs in combination with chemotherapy or immunotherapy have dramatically improved prognosis in adult Ph+ ALL and are now being assessed in children and adolescents [265, 266]. These results are prompting clinicians to redefine the role of allogeneic HSCT in this disease [267].

**Hypereosinophilic syndrome (HES) and related myeloid/lymphoid neoplasms with** ***PDGFR*** **fusion genes**: Eosinophilic myeloproliferative disorders are characterized by molecular heterogeneity, with rearrangements between two genes that combine together creating active fusion kinases [268]; these kinases constitute clear, targetable mechanisms for this group of disorders [269–271]. As previously mentioned, imatinib targets members of the PDGFR family as well as ABL1. In *PDGFRA/PDGFRB* rearranged HES and related neoplasms, daily imatinib (100 to 400 mg) consistently induced response rates above 90% at both the blood and molecular level [269, 272–276]. Treatment could even be stopped in a substantial number of patients with durable responses, with long-lasting molecular remission maintained off therapy [277, 278]. Imatinib may even be effective in a subset of *PDGFRA/PDGFRB*-negative HES cases, especially when the disease appears clonal [279]. This also strengthens the concept that eosinophilic disorders should be molecularly classified, as a single genetic fusion event can define prognosis and optimal therapy [280].

**Systemic mastocytosis (SM):** SM is a rare hematological neoplasm, characterized by abnormal proliferation of tissue mast cells. Most cases are driven by activating KIT variants, especially KIT D816V, which changes the kinase tridimensional shape and makes it relatively resistant to imatinib [281, 282]. As a result, imatinib is mainly used only when rare non-D816V *KIT* mutations are present, while other TKIs (especially midostaurin and avapritinib) that can inhibit D816V have expanded treatment options and reshaped the SM treatment landscape [281, 283, 284].

**Gastrointestinal stromal tumor (GIST):** GIST was one of the earliest solid-tumor proofs that the CML blueprint could work beyond leukemia. Once *KIT-* and *PDGFRA*-activating mutations were identified as key drivers, a disease that had resisted chemotherapy became a kinase-defined entity that could be selectively targeted [285, 286]. Imatinib delivers durable benefit in metastatic GIST, making molecular stratification central to treatment in this solid tumor [286, 287]. GIST also mirrored CML’s evolutionary story: resistance often arose through secondary *KIT* mutations, supporting stepwise use of additional TKIs and showing that resistance can guide the next step of drug design [288, 289].

**Unresectable, recurrent and/or metastatic dermatofibrosarcoma protuberans (DFSP) with** ***PDGFB*** **fusion:** DFSP is a rare cutaneous tumor often associated with the fusion of the *PDGFB* and collagen 1A1 (*COL1A1*) genes [290, 291]. Although treatment is often surgical, unresectable, recurrent and/or metastatic tumors have been successfully treated with imatinib [292], which is now the recommended therapy in this setting [293].

In summary, CML left a clear pathway that oncology now follows: find the driver, block it selectively, and monitor molecular response over time [255]. Although solid tumors introduced additional complexities, including increased heterogeneity, microenvironment effects, and parallel signaling pathways, the same conceptual toolkit from CML was successfully carried over.

## Conclusions

Imatinib was approved for CML treatment in 2001. By 2010, the first TFR trial showed that a substantial proportion of patients could discontinue imatinib treatment and remain disease free. Thus, the prospects for CML patients changed dramatically within less than 10 years, from an almost certain death sentence to a functional and potentially molecular cure. In the era of TKI therapy, patients with CML can anticipate a near-normal life expectancy provided they can access and afford TKIs, comply with therapy, are monitored optimally, their AEs are managed to achieve good QoL, and their later-line therapies are implemented promptly at the earliest indication of CML resistance.

Words like transformative and radical cannot convey the significance of the impact imatinib and other TKIs have had on the lives of patients with CML and other malignancies. Numbers may express this better: CML is a rare disease, affecting approximately 1 in 100,000 people per year, but its prevalence is expected to grow 10-fold because the mortality rate is so low [106]. And stemming from this extraordinary impact on survival, another great achievement of TKIs: massive improvement in patient QoL, both from reduced toxicity compared to early therapies and administration in the outpatient setting. The success of TKIs ushered in a new era of patient-centric care that quickly spread to other cancers and disease types. Challenges remain, but novel agents promise to address them and the future looks exciting.

It is true that CML was the perfect candidate for a novel targeted therapy: a cancer driven by a single mutation, identified and thoroughly characterized so that the challenges that rose along the way could be solved relatively easily thanks to amassed knowledge on the biology of the disease. Thus the story of TKIs is a story of collaboration, between geneticists, chemists, biologists, clinicians and other healthcare professionals. It is also a story of hope trumping doubt, because despite the known basis of disease and the elegance of the approach, nothing like this had ever been attempted before and a seismic shift in mindset was needed to bring TKIs into clinical trials. Once again, we can let numbers speak for themselves: the 100^th^ TKI was approved in 2025 by the FDA [294]. TKI targets now include autoimmune and pulmonary diseases, among others, as well as various solid tumors such as breast cancer, colorectal cancer, melanoma, non-small cell lung cancer, thyroid cancer, and renal cell carcinoma.

The 25 years that have lapsed since the approval of imatinib have been tremendously exciting for the treatment of cancer and other diseases; the next 25 years will likely be equally or more exciting.
